# Intraoperative visualization of a deformed left main stent during surgical aortic valve replacement

**DOI:** 10.1186/s13019-023-02120-9

**Published:** 2023-01-31

**Authors:** Philipp P. Müller, Christian Heim, Michael Weyand, Frank Harig

**Affiliations:** Department of Cardiac Surgery, Friedrich Alexander University Erlangen-Nuremberg, 91054 Erlangen, Germany

**Keywords:** Cardiac surgery, Aortic valve replacement, Coronary artery disease, Left main stenosis, Stenting, Left main stenting, Cardiology

## Abstract

**Background:**

While coronary artery bypass grafting is typically considered first choice for the treatment of left main stenosis, there is a trend towards left main stenting due to a steadily aging population in western countries with a high operative risk and patients with single vessel coronary artery disease affecting the left main artery. Nevertheless left main stenting remains controversial, especially in patients with concomitant indications for open-heart surgery.

**Case presentation:**

We want to present a case of a 78-year-old male patient with high-grade aortic stenosis who underwent surgical aortic valve replacement at our heart center due to anatomical contraindications for transcatheter aortic valve replacement. Stenting of the left main coronary artery was performed three years earlier due to single vessel coronary artery disease while moderate aortic valve stenosis was under surveillance at the time of the intervention. Intraoperatively we found the stent to be deformed inside the left main coronary artery, covering nearly 25% of the coronary ostium. So injection of cardioplegia directly into this ostium, as we perform normally, was not possible without further damaging the stent and/or the opening of the ostium. We had to insert cardioplegia via the retrograde way, so via the coronary sinus.

**Conclusion:**

While left main stenting can be reasonable for a specific population of patients, it should be used cautiously in patients with concomitant indications for open-heart surgery in the near future and a low perioperative risk profile.

## Background

Left main (LM) coronary artery stenosis is found in up to 7% of patients undergoing coronary angiography [1] and treatment of these lesions is crucial as the risk of mortality for untreated LM-stenosis is high. While surgical revascularization is considered the treatment option of choice for LM-stenosis [2], there is an ongoing discussion about the feasibility of LM-intervention via stenting. This paradigmatic change is precipitated by advances in the development of stents over the past years and many investigational studies reporting safety and efficacy of left main stenting with both bare metal (BMS) and drug eluting (DMS) stents [3]. Nevertheless left main stenting bares a risk of abrupt closure and re-stenosis, which are fatal complications for patients.

CAD and aortic stenosis (AS) are conditions that commonly coexist, especially in the elderly. While European guidelines clearly recommend CABG for patients with coexisting severe aortic stenosis, requiring aortic valve replacement (AVR) [4], there are no specified recommendations for patients with moderate aortic stenosis and concomitant CAD with LM- stenosis due to lacking data.

The purpose of this report is to point out the need for precise recommendations for the treatment of patients with left main disease and concomitant moderate aortic valve stenosis to improve the outcome and to help standardize therapeutic decisions in heart teams.

## Case presentation

**Fig. 1:**
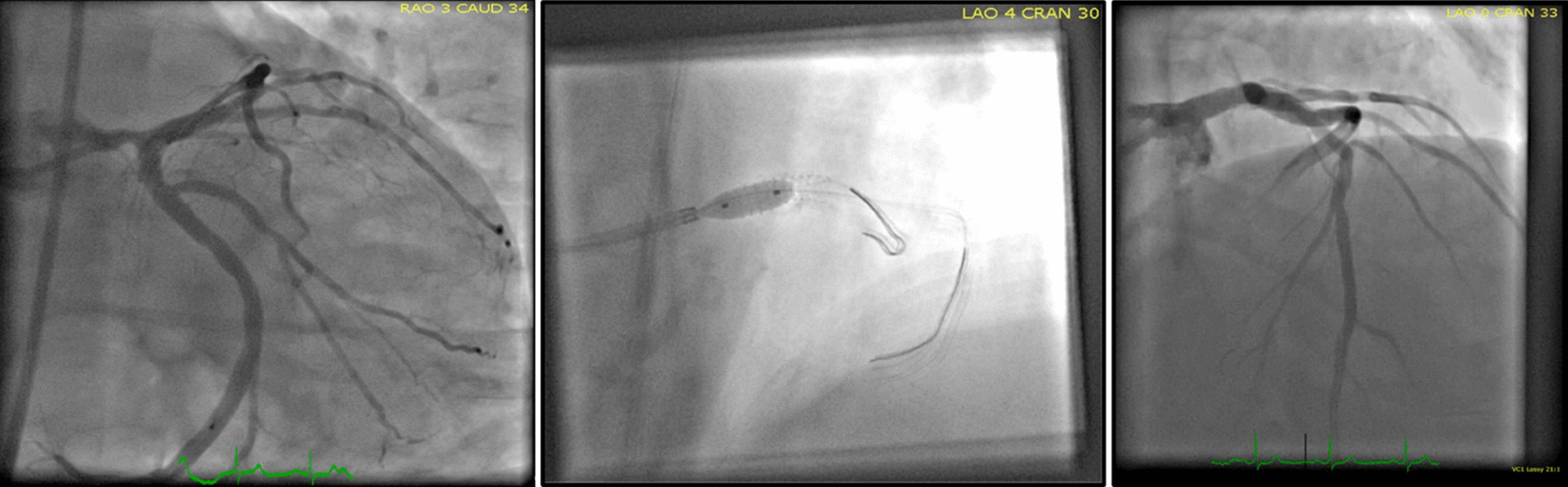
LM-Stenting with Kissing Balloon technique in 2017

A 78-year old male patient was admitted to our hospital from an external clinic with intermittent shortness of breath and chest pain. The patient’s history included coronary artery disease with a LM-Stenosis of 75% and previously performed left main stenting in 2017. The stenting was performed as an elective procedure when CAD with LM-involvement was diagnosed in 2017 using intravascular ultrasound and a POT and kissing balloon technique (Xience Alpine-Stent 4.0/15 mm, Fig. 1). Additionally the patient was suffering from moderate aortic stenosis, arterial hypertension and obesity. At the time of stenting, the aortic valve stenosis showed a Vmax of 3.1 m/s, the gradient was 40/23 (dpmax/dpmean), the iAVA was 1.1 cm^2^/2.09 BSA = 0.52 with with a reduced ejection fraction of 45% and a severe LV-hypertrophy (IVS: 14 mm).

**Fig. 2:**
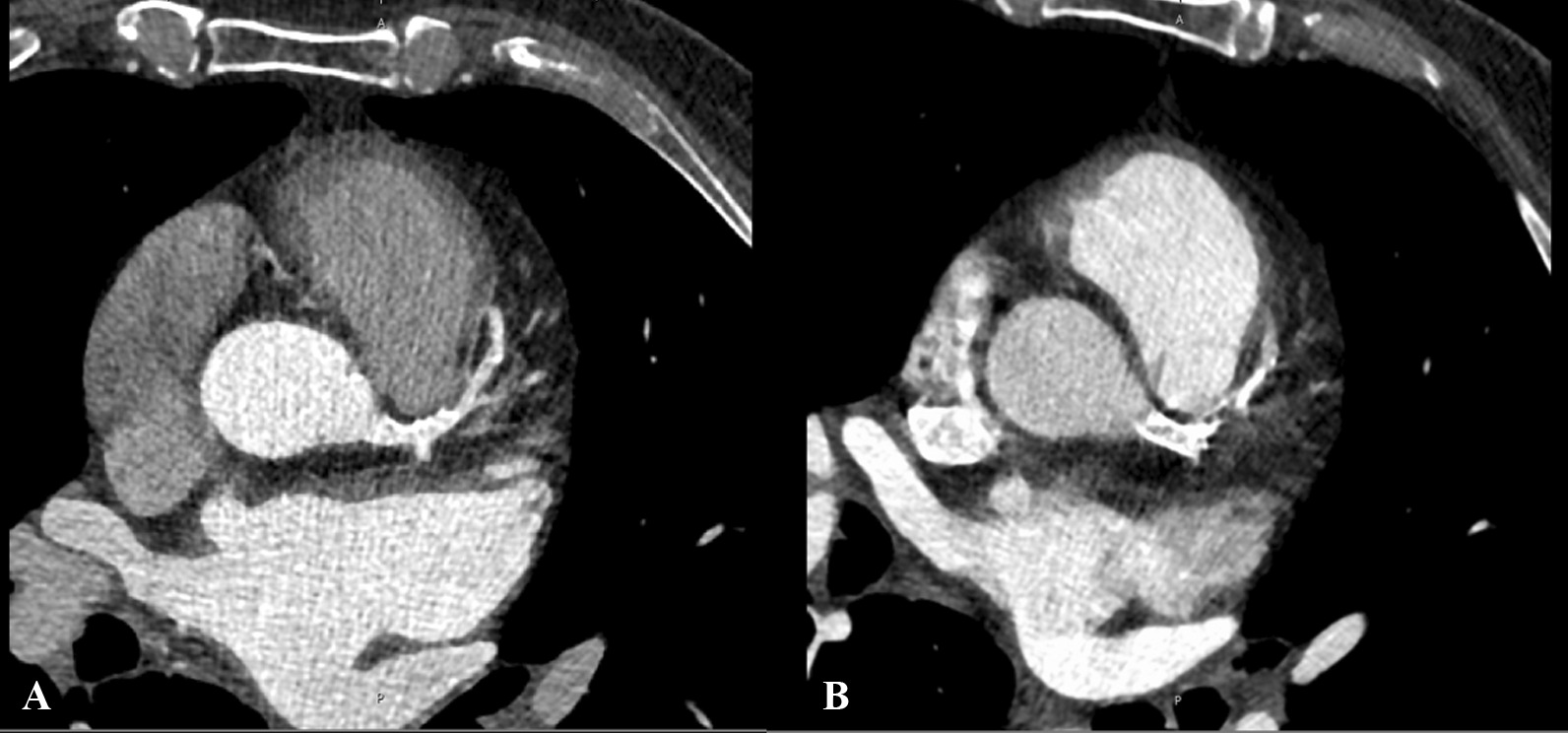
CT-Scan in 2017 **A** and 2021 **B** (CT-Siemens—SOMATOM Force)

Physical examination, electrocardiography and blood levels showed no abnormalities at admission. Coronary angiography was performed but showed no stenotic lesions of the coronary arteries. In the echocardiographic assessment, a high-grade aortic stenosis with an aortic valve orifice area of 0.6 cm^2^ and a reduced ejection fraction (EF) of 40% was found. In accordance to current guidelines the patient’s case was discussed in the clinic´s heart team. To complete diagnostics, computer tomography of the heart and the aorta was performed showing an aortic annulus of 7.56 cm^2^ (Fig. 2) and a bicuspid aortic valve. Due to these conditions, the patient was scheduled for surgical aortic valve replacement (SAVR) at the department of cardiac surgery.

**Fig. 3:**
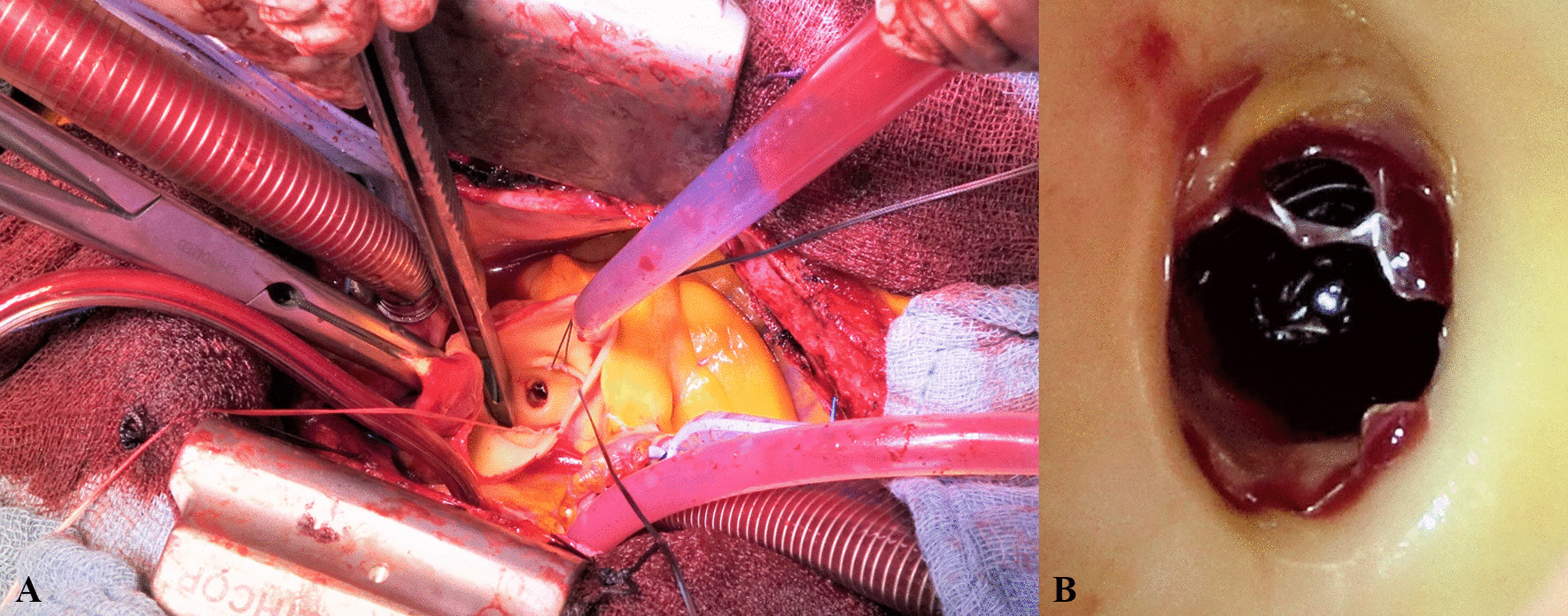
Intraoperative visualization of a deformed left main stent (**A** Intraoperative situs; **B** Left main stent covering nearly 25% of the left coronary ostium)

SAVR was performed with a minimally invasive approach via right—J-mini-sternotomy. After establishing cardiopulmonary bypass, blood-cardioplegia was applied ante- and retrograde for myocardial protection. Subsequently aortotomy was performed showing a heavily calcified, bicuspid aortic valve, which was excised and replaced with a 27 mm biological prosthesis. When cardioplegia was applied directly through the left coronary ostium, we noticed the previously implanted Stent to be deformed, covering nearly 25% of the coronary ostium (Fig. 3).

Following the procedure, the patient was relocated to the ICU. Further clinical course was uneventful and the patient was discharged home 10 days after surgery in good condition.

## Discussion

Studies comparing CABG vs. PCI for the treatment of LM-stenosis have shown similar safety and mortality rates for both procedures [5]. On the other hand studies implicated a higher rate of repeat revascularization after LM-stenting which in terms is associated with a higher morbidity for affected patients [6] while data from experimental models have shown that stents, placed at sites in the coronary system where continuous stress occurs like at the coronary ostium, are more prone to deform over time [7] which can result in fatal complications for patients. Additionally, LM-stenting is supported by both European and American guidelines only for a specific population of patients with a low SYNTAX score and both guidelines strongly recommend to consult the heart team for the decision making process [4, 8]. Recently published guidelines from the European Society of Cardiology (ESC) and the European Association for Cardio-Thoracic Surgery (EACTS) clearly indicate that patients with CAD requiring valve interventions at the same time in whom CABG is indicated, benefit from concomitant SAVR [9]. According to the aforementioned guidelines SAVR should be considered in patients with moderate aortic stenosis in which surgical revascularization is indicated. The guidelines for the management of patients with valvular heart disease by the American college of cardiology and the American heart association recommend SAVR and CABG for patients with a significant aortic stenosis and concomitant LM-stenosis, with a reduced risk for perioperative myocardial infarction in affected patients [10]. Table 1 summarizes current recommendations for patients with valvular heart disease and concomitant CAD from both European and American guidelines. In this case the patient was an eligible candidate for CABG and SAVR in 2017 when both aortic valve- and LM-stenosis where diagnosed according to ESC/EACTS guidelines. Nevertheless more studies are required to evaluate standardized treatment strategies for patients with moderate aortic valve stenosis and left-main-disease as a case-by case-decision indicated by those guidelines [4] might be to the disfavor for patients as described in this case. Coronary artery bypass grafting remains the treatment option of choice for patients with left main stenosis especially in those with a low operative risk and other indications for heart surgery. Clear recommendations for patients with left main stenosis and coexisting moderate aortic valve stenosis are required in future guidelines to improve the long-term outcome for affected patients. At the same time LM- stenting is often performed without respect to possible surgical operations in the near future which makes a heart team approach an important part of the decision making process. In this case consideration of guidelines for the treatment of valvular heart disease at the time of revascularization may have led to a more definitive solution for the patient, favoring CABG and SAVR over PCI. Future guidelines could emphasize a more detailed approach regarding standardized procedures for patients with concomitant heart disease especially in terms of moderate aortic stenosis as described in this case.

**Table 1:** AHA/ACC and ESC/EACTS Guidelines for Patients with CAD and AS

Guideline	Recommendation	Class/level of evidence
AHA/ACC	Patients undergoing SAVR with significant proximal CAD CABG is reasonable for selective patients AND in patients with significant AS and CAD SAVR and CABG is preferred over TAVI and PCI	IIa/C
ESC/EACTS	SAVR should be considered in patients with moderate Aortic stenosis undergoing CABG or surgical intervention on the ascending aorta or another value after Heart Team discussion	IIa/C

## Conclusion

Although LM-stenting has become a widespread intervention in recent years, it should be used cautiously in patients when open procedures are likely to be necessary in the future. While LM-stenting might be reasonable for some patients, it should be used cautiously in patients with concomitant indications for open-heart surgery in the near future and a low perioperative risk profile.

## Data Availability

Not applicable.

## Abbreviations

ACC: American college of cardiology
AHA: American heart association
AS: Aortic stenosis
AVR: Aortic valve replacement
BMS: Bare metal stents
CABG: Coronary artery bypass grafting
CAD: Coronary artery disease
CT: Computed tomography
EACTS: European association for cardio-thoracic surgery
EF: Ejection fraction
ESC: European society of cardiology
ICU: Intensive care unit
IVS: Interventricular septum
LM: Left main coronary artery
LV: Left ventricular
PCI: Percutaneous coronary intervention
POT: Proximal optimization technique
SAVR: Surgical aortic valve replacement
